# IL-12p40 is essential but not sufficient for *Francisella tularensis* LVS clearance in chronically infected mice

**DOI:** 10.1371/journal.pone.0283161

**Published:** 2023-03-27

**Authors:** Lara R. Mittereder, Jonathan Swoboda, Roberto De Pascalis, Karen L. Elkins

**Affiliations:** Center for Biologics Evaluation and Research, Food and Drug Administration, Silver Spring, Maryland, United States of America; University of Northern Colorado, UNITED STATES

## Abstract

IL-12p40 plays an important role in *F*. *tularensis* Live Vaccine Strain (LVS) clearance that is independent of its functions as a part of the heterodimeric cytokines IL-12p70 or IL-23. In contrast to WT, p35, or p19 knockout (KO) mice, p40 KO mice infected with LVS develop a chronic infection that does not resolve. Here, we further evaluated the role of IL-12p40 in *F*. *tularensis* clearance. Despite reduced IFN-γ production, primed splenocytes from p40 KO and p35 KO mice appeared functionally similar to those from WT mice during *in vitro* co-culture assays of intramacrophage bacterial growth control. Gene expression analysis revealed a subset of genes that were upregulated in re-stimulated WT and p35 KO splenocytes, but not p40 KO splenocytes, and thus are candidates for involvement in *F*. *tularensis* clearance. To directly evaluate a potential mechanism for p40 in *F*. *tularensis* clearance, we reconstituted protein levels in LVS-infected p40 KO mice using either intermittent injections of p40 homodimer (p80) or treatment with a p40-producing lentivirus construct. Although both delivery strategies yielded readily detectable levels of p40 in sera and spleens, neither treatment had a measurable impact on LVS clearance by p40 KO mice. Taken together, these studies demonstrate that clearance of *F*. *tularensis* infection depends on p40, but p40 monomers and/or dimers alone are not sufficient.

## Introduction

*Francisella tularensis*, an intracellular bacterium that causes tularemia, continues to present a public health threat in some areas of the world [1]. *F*. *tularensis* is categorized as a potential agent of bioterrorism due to its ability to cause fatal disease through airborne transmission at a low infectious dose [2]. *F*. *tularensis* Live Vaccine Strain (LVS), an attenuated strain developed from *F*. *tularensis* subsp. holarctica (Type B), provides some protection against tularemia in rodents and humans [3, 4]. Although LVS is not licensed in the United States, it has been studied in people as an investigational vaccine [5]. Moreover, LVS is a useful tool in rodent models to study primary and secondary immunity against fully virulent *F*. *tularensis* [6]. In animals, long term protection provided by LVS is heavily dependent on the development of robust Th1 responses. T-cell derived TNF-ɑ and IFN-γ are key components in immunity to LVS; both are induced by the production of IL-12 early in primary and secondary infection [7].

IL-12 therefore plays a central role in cell-mediated immunity by promoting and maintaining Th1 responses, which are crucial for protection against many intracellular pathogens, including *F*. *tularensis*. Mice deficient in IL-12 are more susceptible to infection with intracellular pathogens such as *Leishmania major*, *Toxoplasma gondii*, *Cryptococcus neoformans*, and *Mycobacterium tuberculosis* [8–11]. Upon activation by these infections, antigen presenting cells produce IL-12 that stimulates IFN-γ production from T cells and natural killer (NK) cells [12]. IL-12 also upregulates the expression of its receptor (IL-12R) on activated T cells, B cells, and NK cells, which further potentiates IFN-γ production. Decreased protection in the absence of IL-12 in many of these studies has correspondingly been attributed to reduced availability of IFN-γ [8, 13, 14].

Commonly designated IL-12p70, complete IL-12 is a heterodimeric cytokine composed of a p40 and a p35 subunit, each expressed by separate genes on different chromosomes [12]. In contrast to p35, which is produced at lower levels and is not secreted, IL-12p40 is produced and secreted in excess during activation. Further, IL-12p40 binds to other subunits to form additional members of the IL-12 family. IL-23, a heterodimer of p40 and a distinct monomer denoted p19, binds to a separate receptor (IL-23R) to induce proliferation and IL-17 production by memory T cells.

In addition to its role as components of IL-12p70 and IL-23, other evidence indicates that p40 as a monomer or homodimer (p40 or p80, respectively) can interact with its receptor IL-12Rβ1 to exert agonistic and/or antagonistic functions that are distinct from those of its other family members. These functions are not as well understood, particularly in the context of infectious disease. Early *in vitro* experiments showed that p80, and to a lesser extent p40, blocked IL-12p70 activity in concanavalin A-activated mouse lymphocytes; further, p80 inhibited IFN-γ production in mouse splenocytes treated with IL-12p70 [15]. On the other hand, subsequent studies suggested immunostimulatory roles for p80 in promoting dendritic cell (DC) migration during mycobacterial infection in mice [16]. Similarly, p80 may exacerbate disease during asthma and viral infections in mice and humans, in part by promoting macrophage migration into the lungs [17–19].

Independent activities of p40 monomer are much less clear. Although p40 was once thought to be inert, *in vitro* studies have shown that mouse microglial and peritoneal macrophages stimulated with recombinant p40 produce TNF-α in a dose-dependent manner, and to a similar degree as cells stimulated with recombinant p80 or p70 [20]. Abdi *et al*. proposed that p40 monomer may serve as a DC-derived probe to sample the composition of the local milieu by looking for extracellular binding partners, which would then enable communication to cells downstream of DC activation such as NK cells and T cells [21].

Importantly, IL-12 plays a complex role in the clearance of LVS during vaccination. Mice treated *in vivo* with neutralizing antibodies against IL-12 survive high doses of primary and secondary LVS infection but are ultimately unable to clear the bacteria [22]. The roles of each IL-12 subunit were directly demonstrated using the respective KO mice: IL-12p40 KO mice never clear even a low dose intradermal (i.d.) or intranasal (i.n.) LVS infection, while IL-12p35 KO mice display only a slight delay in bacterial clearance, despite low IFN-γ levels in both KO animals [22, 23]. Subsequent studies using IL-23p19 KO mice revealed that IL-23 is generally dispensable for resistance and clearance of both i.d. and i.n. LVS infection, ruling out IL-23 as part of the mechanism of clearance [23]. Immune lymphocytes from LVS-vaccinated p40, p35, and p19 KO mice controlled intracellular LVS growth in *in vitro* co-culture assays similarly to those from wild type (WT) mice, again despite reduced IFN-γ production by both [22, 23], suggesting that all mice generated effective T cell-mediated responses against LVS. Collectively, therefore, the results to date point to an unknown role for IL-12p40 in LVS clearance that is independent of its functions through IL-12p70 or IL-23, and not necessarily reliant on its ability to induce IFN-γ production. Here, we further evaluated the complex role of p40 in *F*. *tularensis* infection clearance. We focused on the hypothesis that IL-12p40 itself effects clearance, and we tested this possibility reconstituting p40 using multiple methods.

## Materials and methods

### Experimental animals

Male C57BL/6J, B6.129S1-*Il12b*^tm1Jm^/J (referred to here as p40 KO mice), and B6.129S1-*Il12a*^tm1Jm^/J mice (referred to as p35 KO mice) were acquired from Jackson Laboratories (Bar Harbor, ME) and housed in sterile microisolator cages at CBER/FDA. All animals had access to sterilized food and water *ad libitum*. All procedures were performed under protocols approved by the Animal Care and Use Committee of FDA, which reviews and approves proposed animal numbers associated with experimental protocols. The health of all animals, including that of infected animals, is monitored daily by professional animal caretaking staff. Mice are evaluated for visible symptoms, weight loss, and unexpected behaviors. No morbidity was expected or observed in these studies, but any mice that exhibit more than 20% weight loss, pronounced hunched posture, or are unable to move sufficiently to obtain water or food are humanely euthanized by CO_2_ inhalation within 1 hour per CBER standard operating procedure. Animals euthanized at pre-determined endpoints based on previous data and per experimental design were humanely euthanized by CO_2_ inhalation per CBER standard operating procedure. Animal handling and euthanasia were performed by appropriately trained staff according to approved animal protocols. Experimental mice were age-matched within an experiment at the ages indicated in figure legends.

### Bacteria and growth conditions

*F*. *tularensis* LVS (ATCC 29684) was used to prepare bacterial stocks in modified Mueller Hinton (MH) broth as previously described [24]. In brief, LVS from ATCC was streaked on MH plates for colony isolation. A single colony was expanded and grown to mid-log phase in broth in a shaking incubator, then aliquoted and stored at -80°C. Concentrations of live bacteria were determined through plating for colony forming units (CFU). As a quality control measure, the expected LD_50_ and time to death for each new bacterial stock was confirmed through i.p. injections in male BALB/cByJ mice [25].

### *In vivo* bacterial infections

Mice were infected with the indicated dose of LVS through the i.d. route in 0.1 ml sterile PBS (Lonza, Basel, Switzerland) and euthanized at the time points indicated in the figure legends. True doses were determined by plate count of the diluted inoculum. Bacterial burdens of infected mice were determined by CFU plate counts of organ homogenates or whole blood. After euthanasia, blood was collected by cardiac puncture and spleen, liver, and lungs were aseptically removed. Spleen, liver, and lungs were homogenized in 3–5 ml sterile PBS using a Stomacher (Seward, West Sussex, UK). Homogenates and whole blood were plated on MH plates at appropriate dilutions. Sera were prepared from whole blood using serum gel microtubes (Sarstedt, Nümbrecht, Germany). Homogenates and sera were stored at -80°C for later analyses.

### Preparation of splenocytes

Spleens were aseptically removed and transferred to a sterile culture plate containing PBS supplemented with 2% FBS. Spleens were then homogenized using the plunger of a 3 ml sterile syringe, and the homogenates were passed through a 40 μM cell strainer. Homogenates were treated with ACK lysis buffer to remove red blood cells and washed. An aliquot of the single cell suspension was stained with trypan blue to assess cell viability and enumerated using a hemocytometer.

### *In vitro* co-culture assays

Co-cultures of bone marrow-derived macrophages (BMDM) and splenocytes were performed as previously described [26]. Briefly, BMDM were cultured for 6–7 days to a confluent monolayer in 24-well or 48-well plates in Dulbecco’s modified Eagle’s medium (Lonza) supplemented with 10% serum-inactivated fetal bovine serum (Hyclone, Logan, UT), 10 ng/ml mouse M-CSF (PeproTech, Rocky Hill, NJ), 0.2 mM L-glutamine, 0.1 mM nonessential amino acids, 10 mM HEPES buffer, 1 mM sodium pyruvate, and 1 mM sodium bicarbonate. BMDM were infected with *F*. *tularensis* LVS at a multiplicity of infection (MOI) of 1:10 (bacterium to BMDM) for 2 hours, after which the monolayer was washed and treated with 50 μg/ml gentamicin for 45 minutes. The cells were then washed, and splenocytes were added to the infected BMDM at a ratio of 1:2 (splenocytes to BMDM) or as indicated in figure legends. After 2 and 3 days, non-adherent cells were recovered and stored in RNA*later* (Invitrogen, Waltham, MA) at -80°C for gene expression analyses. Supernatants were also saved at -80°C for cytokine analyses. Remaining BMDM were lysed and plated for CFU as previously described [26]. Additional cultures were lysed the day of infection to assess bacterial uptake.

### Cytokine and nitric oxide measurements

Organ homogenates, serum samples, and culture supernatants were assayed for IFN-γ, IL-12p40, IL-12p70, TNF-α, and GM-CSF using standard sandwich ELISAs according to manufacturer’s instructions (BD Biosciences, Franklin Lakes, NJ). Optical density was read at 405 nm on a VersaMax microplate reader (Molecular Devices, San Jose, CA). Cytokine concentrations were quantified by comparison to known concentrations of the applicable recombinant standard (BD Biosciences) using four-parameter fit regression in SOFTmax Pro ELISA software. Nitric oxide was measured using a Griess reaction kit according to manufacturer’s instructions and quantified by comparison to a serially diluted standard (Molecular Probes, Eugene, OR).

### Western blots

Proteins from HEK293T cells were isolated in RIPA buffer with protease inhibitors (Sigma-Aldrich, St. Louis, MO) and quantified using Pierce 660 nm Protein Assay Reagent against a BSA standard, following the manufacturer’s protocol (Thermo Scientific, Waltham, MA). Reduced (+β-ME) and non-reduced samples were separated on a 4–20% mini-PROTEAN TGX gel (Bio-Rad, Hercules, CA). Proteins were transferred to a nitrocellulose membrane using an iBlot 2 Transfer system (Invitrogen), according to manufacturer’s protocols. Membranes were blocked with 5% BSA in TBST for 1 hour at room temperature, then incubated in a 1/1500 dilution of rat anti-mouse IL-12p40 Ab (C15.6, BD PharMingen, San Diego, CA) in TBST overnight at 4°C. After washing, membranes were incubated with a 1/1000 dilution of rabbit anti-rat IgG alkaline-phosphatase-conjugated Ab (Southern Biotechnology, Birmingham, AL) in TBST for 1 hour at room temperature. Membranes were developed for 10 minutes using BCIP/NBT phosphatase substrate (SeraCare Life Sciences, Gaithersburg, MD) and scanned using a high-resolution scanner.

### Gene expression profiling

For splenocytes recovered from co-cultures, in each experiment cells from three wells of a 24 well plate were pooled for each experimental group. Total RNA was extracted from pooled cells stored in RNA*later* using the RNeasy Mini Kit (Qiagen, Hilden, Germany), and cDNA was synthesized (High Capacity RNA-to-cDNA Kit, Applied Biosystems, Waltham, MA) according to manufacturer’s instructions. Gene expression was analyzed by semi-quantitative RT-PCR using a ViiA 7 sequence detection system (Applied Biosystems) with Th1-Th2-Th3 RT^2^ Profiler PCR Array cards (Qiagen) containing a pre-determined set of primers for 84 inflammatory cytokine and chemokine genes and 12 housekeeping genes and internal controls. Within each experiment, an average of the housekeeping genes Gusb, Hprt1, Hsp90ab1, Gapdh, and β-actin was used to normalize the data. Normalized data was then used to calculate delta Ct (ΔCt) and the ratio between ΔCt of infected samples and naïve control samples within each genotype.

### Preparation of lentiviral vector

The lentiviral plasmid construct pHAGE-CMV-MCS-IRES-ZsGreen-W was acquired from Addgene (Watertown, MA) and modified to create an IL-12p40 overexpression construct. All plasmid amplifications were performed by transforming One Shot Stbl3 *E*. *coli* (Invitrogen), passaging the bacteria in LB broth with carbenicillin (Sigma Aldrich), and purifying the plasmid with PerfectPrep Endofree Maxi kit (5 Prime, South San Francisco, CA). Murine Il-12b cDNA from splenocytes was amplified by PCR using the KOD Hot Start Polymerase kit (Novagen, Birmingham, UK) with in-house primers that incorporated Notl and BamH1 restriction sites on opposite ends of the amplified region without disruption of the coding sequence. Amplified IL-12b cDNA was analyzed by gel electrophoresis, and the appropriate band was purified and sequenced to confirm that the product matched the previous published sequence [27]. A purified IL-12b cDNA and pHAGE construct was digested with restriction enzymes Notl and BAMH1 (Thermofisher Scientific, Waltham, MA) and ligated according to manufacturer’s instructions. The correct insertion was confirmed with gel electrophoresis of restriction digested plasmid and by sequencing. The vector contained a sequence for the fluorescent marker ZsGreen, in addition to a VSV-G envelope that allows for stability and tropism for several mammalian species, including mice [28]. The resulting lentivirus was replication-incompetent such that after genomic integration, only the internal promotor for the IL-12b gene was active and no further viral replication occurred. pHAGE-CMV-IL12b-MCS-IRES-ZsGreen-W plasmid was packaged into lentivirus by transfecting HEK293T cells with psPAX2 and pMD2.G plasmids along with the constructed plasmid, as described below. Lentivirus-containing medium was collected 48 and 72 hours after transfection and filtered with a low-protein binding 0.45 μm filter. Collected pooled lentivirus was concentrated using Clontech Lenti-X Concentration Solution per manufacturer’s instructions (Mountain View, CA). The concentrated lentivirus pellet was resuspended in sterile PBS. High titer lentivirus used for rescue experiments was produced by Applied Biological Materials, Inc. (BC, Canada).

### Transfection and transduction

Transfection experiments were performed using Xfect Transfection Reagent (Takara Bio, Shiga, Japan) in HEK293T cells (Takara Bio), according to manufacturer’s instructions. Supernatants and transfected cells were collected for analyses 48 hours after transfection.

*In vitro* transduction with lentivirus was performed by seeding 7.5 x 10^5^ HEK293T cells per well in a 6-well tissue culture plate. Approximately 8 hours after seeding, lentivirus was diluted at the indicated concentration in DMEM cell culture media containing 8 μg/mL polybrene (Millipore, Burlington, MA) and added to cells. The plate was then centrifuged for 1 hour at 1200 rpm at room temperature. After overnight culture at 37°C, media containing lentivirus and polybrene was replaced with fresh culture media. Supernatants and transduced cells were collected 48 hours after exposure to lentivirus. Percent ZsGreen^+^ cells were determined by flow cytometry and used to calculate viral titer.

### Recombinant protein and lentiviral treatments

Mice were given 50 μg recombinant murine IL-12p40 homodimer (R&D Systems, Minneapolis, MN) i.p. in 500 μl sterile PBS every other day for 7 days based on previously published data using recombinant p80 in mice [29–31]. Lentivirus was thawed on ice and diluted in sterile PBS to the indicated concentrations and injected i.p. in 500 μl at the timepoints indicated.

### Flow cytometry

Single-cell suspensions of splenocytes were stained for a panel of cell-surface markers and analyzed using a Becton-Dickinson LSRFortessa flow cytometer and FlowJo (BD BioSciences) software as previously described [32]. The following antibodies were used: anti-CD45 (clone 30-F11), anti-TCR-β (clone H57-597), anti-CD19 (clone 1D3), anti-B220 (clone RA3-6B2), anti-CD4 (clone RM4-5), anti-CD8a (clone 53–6.7), anti-CD11c (clone HL3), NK1.1 (clone PK136), anti-CD11b (clone M1/70), and anti-Ly6G (clone 1A8). Antibody concentrations were previously optimized for use in multi-color staining protocols, using a variety of fluorochromes as needed (BD Biosciences or Biolegend, San Diego, CA). Dead cells were excluded using Live/Dead staining kit (Invitrogen). Live CD45^+^ were further gated to remove aggregates by using SSC-W / SSC-H, and FSC-W / FSC-H parameters. A minimum of ten thousand total events were then counted.

### Statistical analyses

Statistical significance of the indicated differences was assessed with Prism GraphPad using the statistical test indicated in figure legends.

## Results

### IL-12p40 KO mice do not clear chronic sublethal *F*. *tularensis* infection

**Fig 1:**
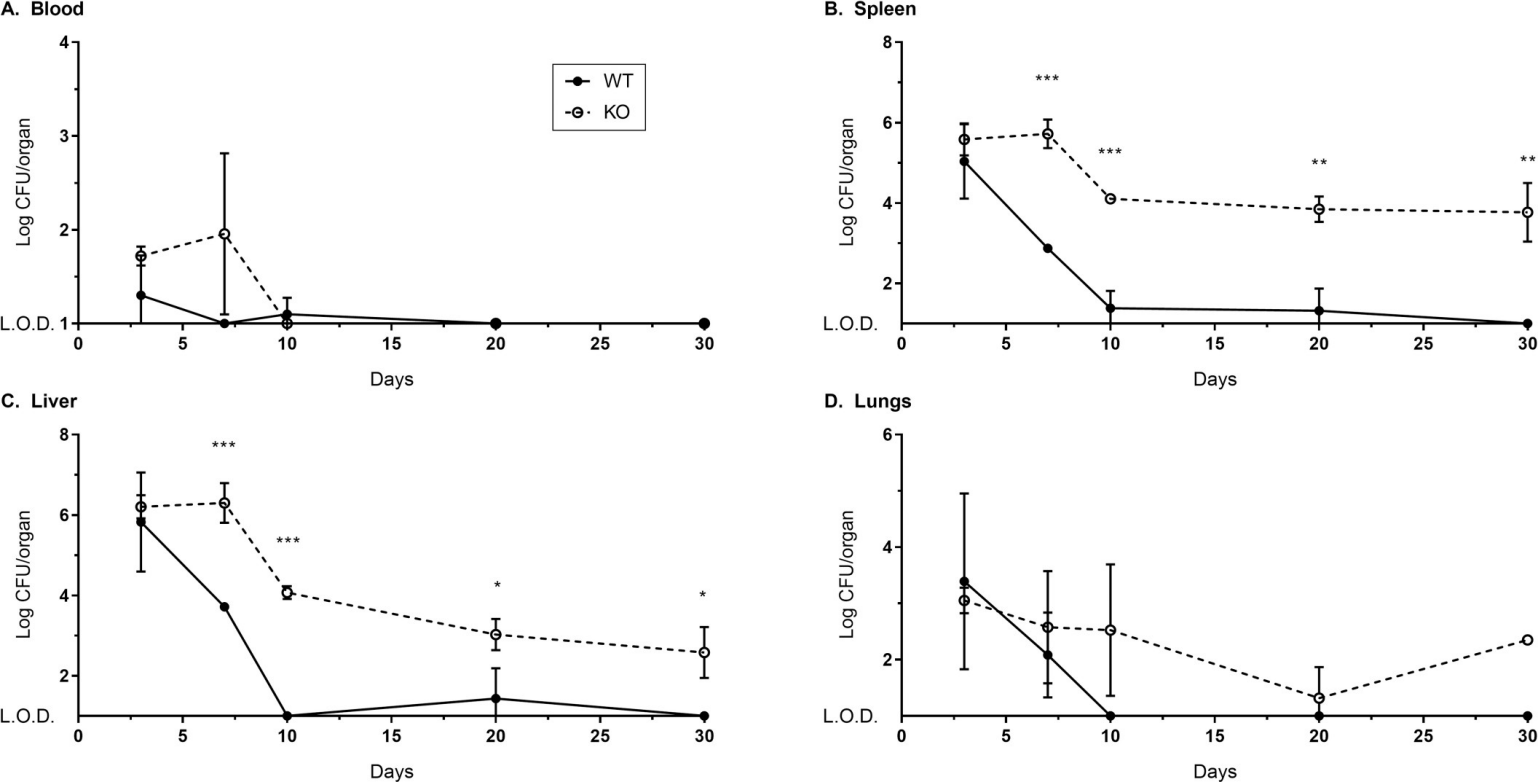
IL-12p40 KO mice maintain chronic LVS infection. Mice were infected with 10^5^ LVS i.d. Three mice per group were sacrificed at 3, 7, 10, 20, and 30 days after infection to assess bacterial burden in blood, spleen, liver, and lungs. Data shown are from a representative experiment of three independent experiments. Mice used were ages 2–6 months and were aged matched within each experiment. *p < 0.05; ** p < 0.01; *** p < 0.001 by Holm-Šídák multiple t-test indicate differences between WT and KO samples at the corresponding time points.

Although wild type (WT) mice clear vaccinating LVS infections from tissues within 2–4 weeks [33], previous data showed substantial bacterial burdens in spleens of p40 KO mice months after infection with low dose *F*. *tularensis* LVS [22]. To confirm previous findings and assess the course of sublethal LVS infection in the blood and additional compartments, we infected WT and p40 KO mice with 10^5^ LVS i.d. and evaluated organ burdens in whole blood, spleen, liver, and lungs throughout a month of infection (Fig 1). While WT and p40 KO mice had comparable CFU after 3 days of infection, by 7 days of infection KO mice had significantly higher bacterial burdens in spleens and livers compared to WT mice, and CFU in whole blood of p40 KO mice trended higher. WT mice controlled and then cleared LVS infection between 10 and 30 days, but p40 KO mice had measurable CFU in spleens, livers, and lungs up to the experimental endpoint at 30 days after infection. Therefore, bacterial burdens decreased after 10 days in p40 KO mice, but they maintained a low chronic infection with LVS. Additional analyses of splenocyte subpopulations by flow cytometry revealed that by 30 days after infection, p40 KO mice had significantly lower proportions of T cells and NKT cells in the spleen compared to WT mice (S1 Fig) and significantly higher proportions of neutrophils and macrophages.

**Fig 2:**
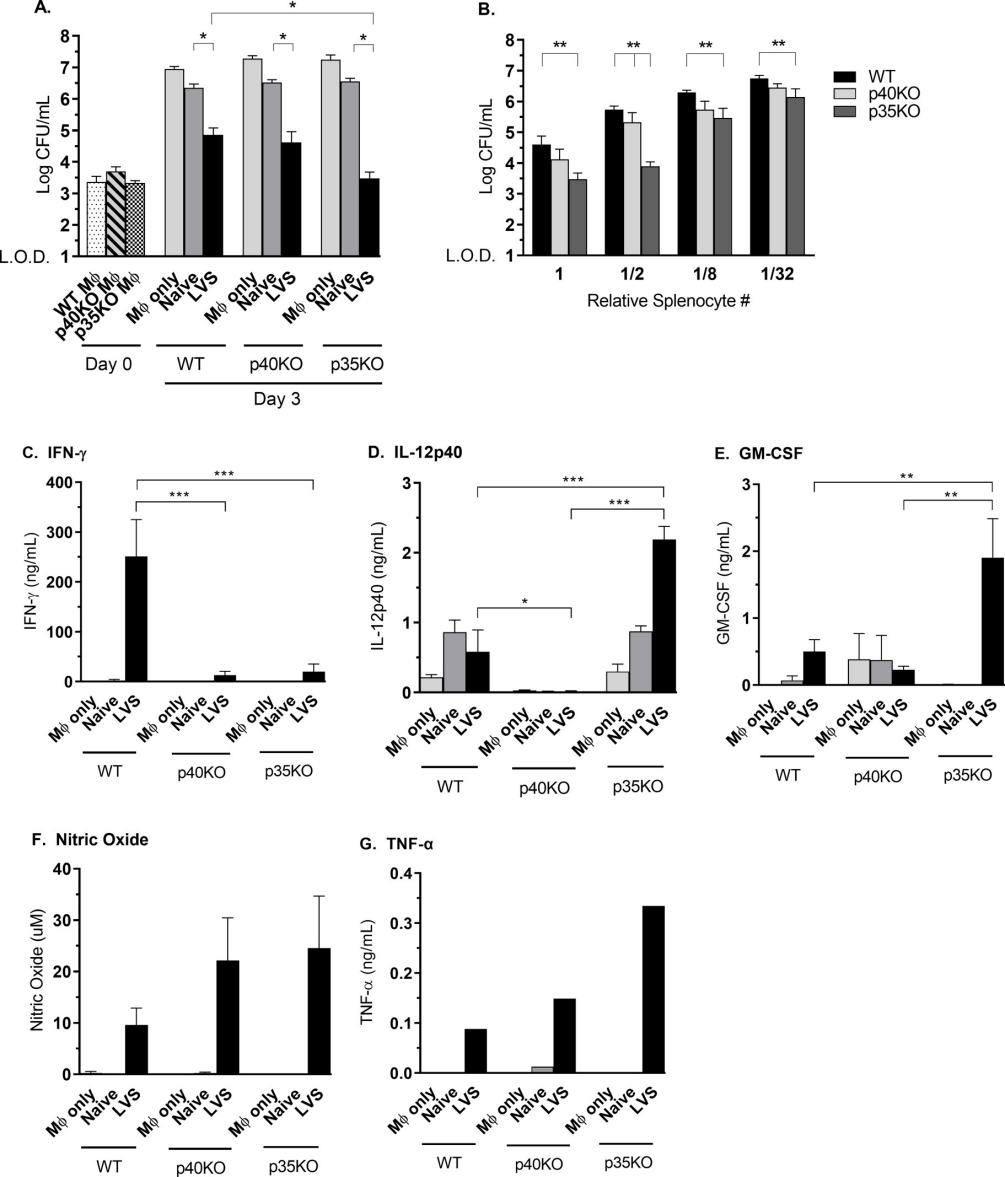
IL-12p40 KO splenocytes control intramacrophage LVS replication. Splenocytes from 5 naïve mice or 5 mice vaccinated with 10^5^ LVS i.d. were co-cultured with LVS-infected BMDMs from the same genotype. Bacterial CFU were assessed immediately after infection (Day 0) and three days after infection (A). Decreasing numbers of splenocytes were added to a constant number of infected BMDMs, where 1 = 5 x 10^6^ splenocytes. Intramacrophage bacterial growth was assessed three days after infection (B). Supernatants pooled from three wells per group were collected on day 3 after infection from the experiment illustrated in (A) for analyses of IFN-γ (C), IL-12p40 (D), GM-CSF (E), nitric oxide (F), and TNF-α (G). Data shown are averages of three independent experiments (A–F) or one experiment (G). Mice used were ages 3–9 months and were aged matched within each experiment. *p < 0.05; ** p < 0.01; *** p < 0.001 by ANOVA with Tukey’s multiple comparisons test.

T cells and T cell-derived cytokines, such as IFN-γ and TNF-α, are essential for complete murine immunity to *F*. *tularensis* [7, 34]. We therefore sought to compare T cell functionality in LVS-vaccinated WT, p40 KO, and p35 KO mice. We previously demonstrated that an *in vitro* co-culture system is a valuable approach to evaluate T cell functions in splenocytes from LVS-primed mice [35]. This *in vitro* assay measures the ability of immune lymphocytes to control intracellular bacterial growth in macrophages and correlates with vaccine-induced immunity to *F*. *tularensis* in rodents [35, 36]. Splenocytes from either naïve mice or LVS-vaccinated mice were overlaid on LVS-infected BMDM of the same genotype. Supernatants were recovered after 3 days of co-culture, and the remaining infected macrophages were lysed to assess bacterial CFU. As expected, LVS-primed splenocytes from WT mice controlled bacterial growth in infected macrophages compared to cultures containing naïve splenocytes or infected macrophages alone (Fig 2A). Despite chronic LVS infection in p40 KO mice *in vivo*, including bacteria in splenocytes prepared from these mice, LVS-primed p40 KO splenocytes controlled intramacrophage bacterial growth to a comparable degree as WT splenocytes. Splenocytes from LVS-primed p35 KO mice, which cleared infection by the time splenocytes were obtained (see Fig 1), consistently exhibited even greater control that that exhibited by LVS-primed WT splenocytes.

To further characterize the relative frequency of functional T cells in each splenocyte population, primed splenocytes were titrated in 2-fold dilutions onto a constant number of LVS-infected macrophages of matching genotype (Fig 2B). Control of intramacrophage bacterial growth was reduced as the number of primed splenocytes decreased; however, at all dilutions, LVS-primed cells from p40 KO and p35 KO mice exhibited comparable or even slightly better control of LVS intramacrophage growth compared to WT primed cells. Indeed, as suggested by results using one amount of splenocytes (Fig 2A), the frequency of splenocytes that effected intramacrophage LVS growth control was higher in p35 KO splenocytes than that in WT splenocytes. This may reflect the higher bacterial burden and greater antigen exposure experienced by p35 KO mice due to delayed LVS clearance (Fig 1; [22]).

Culture supernatants obtained on day 3 from the experiment illustrated in Fig 2A were then analyzed for the presence of cytokines and nitric oxide (NO) produced during co-cultures (Fig 2C–2G). In co-cultures containing WT splenocytes, LVS growth control was accompanied by strong production of IFN-γ and NO, as well as the presence of IL-12p40, GM-CSF, and TNF-α in supernatants. LVS-infected WT and p35 KO macrophages produced small amounts of IL-12p40 that increased in supernatants from co-cultures containing naïve and primed splenocytes; as expected, IL-12p40 was not detected in cultures with p40 KO cells. As previously reported, reduced amounts of IFN-γ but high amounts of nitric oxide (NO) were detected in supernatants from co-cultures containing p40 KO or p35 KO primed splenocytes compared to WT primed splenocyte co-cultures [22]. Co-cultures from p35 KO primed cells also contained higher levels of GM-CSF and TNF-α in comparison to WT and p40 KO cultures, although the importance of these differences is uncertain. Taken together, these data suggest that T cell *in vitro* function, in terms of intramacrophage bacterial growth control, is comparable between splenocytes from WT, p40 KO, and p35 KO mice, despite similar deficits in IFN-γ production from p35 KO and p40 KO primed splenocytes.

### Gene expression profiling reveals dysregulation of IFN-γ-related pathways in IL-12p40 KO mice

To further assess potential differences contributing to the lack of bacterial clearance in p40 KO mice, we evaluated the gene expression profiles of splenocytes recovered from *in vitro* co-culture assays after 48 hours using a panel of 84 cytokine and chemokine genes (S1 Table). Gene expression analyses revealed a subset of genes that were differentially regulated after *in vitro* re-stimulation in WT and p35 KO splenocytes, but not p40 KO cells (Table 1). With the exception of IL-27, all of these genes were upregulated in WT and p35 KO splenocytes compared to levels in p40 KO splenocytes. Not surprisingly, relative expression of IFN-γ exhibited the most dramatic differences, with levels reduced in both p40 KO and p35 KO splenocytes compared to WT cells. Most other genes found in this subset are known to be involved in regulating IFN-γ production in T cells, and several have previously been linked to bacterial clearance of primary infection. Taken together, these data represent a subset of genes which failed to be upregulated as the result of p40 subunit absence and thus are candidates for involvement in LVS clearance.

**Table 1:** Gene expression profiling reveals dysregulation of IFN-γ-related pathways.

	Fold Change		
Probe	WT	p40 KO	p35 KO
**Csf2**	17.92	4.12*^#^	52.03*
**Icos**	2.19	0.87	4.77
**Ifng**	119.56	3.72*^#^	22.23*
**IL12rb2**	5.33	1.71	4.49
**IL13**	19.55	2.96*^#^	21.94
**IL17a**	21.51	0.64*^#^	14.80
**IL18r1**	1.77	0.55	1.49
**IL27**	2.13	11.59*	3.13
**IL2ra**	3.98	0.86	3.62
**IL5**	9.34	0.85	8.71
**IL9**	14.84	0.87*^#^	16.46
**Tbx21**	3.68	2.43	6.21
**Tnfrsf8**	4.12	1.13	3.39

Splenocytes from 3–4 naïve mice or mice vaccinated with 10^5^ LVS i.d., were co-cultured with LVS-infected BMDMs from the same genotype. Splenocytes were collected from co-culture two days after infection and used to purify RNA. Gene expression was analyzed by RT-PCR using probes against a panel of 84 inflammatory cytokine and chemokine genes. Genes shown were significantly different between WT and KO splenocytes and/or displayed a pattern of higher expression in WT and p35 KO splenocytes versus p40 KO splenocytes. Values shown are represented as fold changes over genotype-matched naïve samples. Data are the average fold changes from three independent experiments. Mice used were ages 3–9 months and were aged matched within each experiment.

* p < 0.05 between KO and WT

# p < 0.05 between p40 KO and p35 KO by ANOVA with Tukey’s multiple comparisons test.

### Administration of IL-12p40/p80 protein does not rescue IL-12p40 KO mice from chronic *F*. *tularensis* infection

Because T cell functions in LVS-vaccinated p40 KO mice were not grossly deficient, and screening by gene expression did not uncover obvious candidates for the clearance defect, we next sought to determine whether treatment with IL-12p40 protein could phenotypically rescue p40 KO mice. Chronically infected p40 KO mice were treated with either 50 μg recombinant p40 homodimer (p80) or PBS by intraperitoneal (i.p.) injection every other day for 7 days. IL-12p40 was readily detected in spleen and liver homogenates from KO mice treated with p40 homodimer, in amounts somewhat greater than those in LVS-infected mice treated with PBS (S2 Fig). Nonetheless, a substantial number of bacteria were still present in these organs (S2A Fig). Levels of IFN-γ, TNF-α, and NO were similar between groups of mice.

In this preliminary experiment, treatment with p40 homodimer (p80) was initiated after chronic LVS infection was well established. Because treatment did not have any measurable impact on chronic infection, we next evaluated the possibility that IL-12p40 or p80 may need to be present before infection begins and/or at higher levels. For subsequent *in vivo* experiments we constructed a lentivirus that produces murine IL-12p40 continuously (S3A Fig). Transduction of HEK293T cells with pHAGE-IL12b plasmid-containing lentivirus resulted in expression of high levels of both IL-12p40 and p80 protein in a dose-dependent manner that were detectable in supernatants by ELISA and western blot (S3B–S3D Fig); no levels were detected using empty plasmid vector or non-transduced cells. To confirm p40 protein delivery via lentivirus *in vivo* and to determine an appropriate dosing regimen, p40 KO mice were given varying doses of pHAGE-IL12b lentivirus by i.p. injection and then sera acquired at multiple timepoints between three and 27 days. KO mice given pHAGE-IL12b lentivirus at high titers had detectable levels of IL-12p40 in the sera three days after treatment, and low to undetectable levels at later timepoints (S3E Fig). No p40 was detected by ELISA (limit of detection ~ 66 pg/spleen) in 4 of 4 spleen homogenates at the experimental endpoint. We therefore concluded that large amounts and multiple doses of lentivirus would be required to sustain circulating p40 levels.

**Fig 3:**
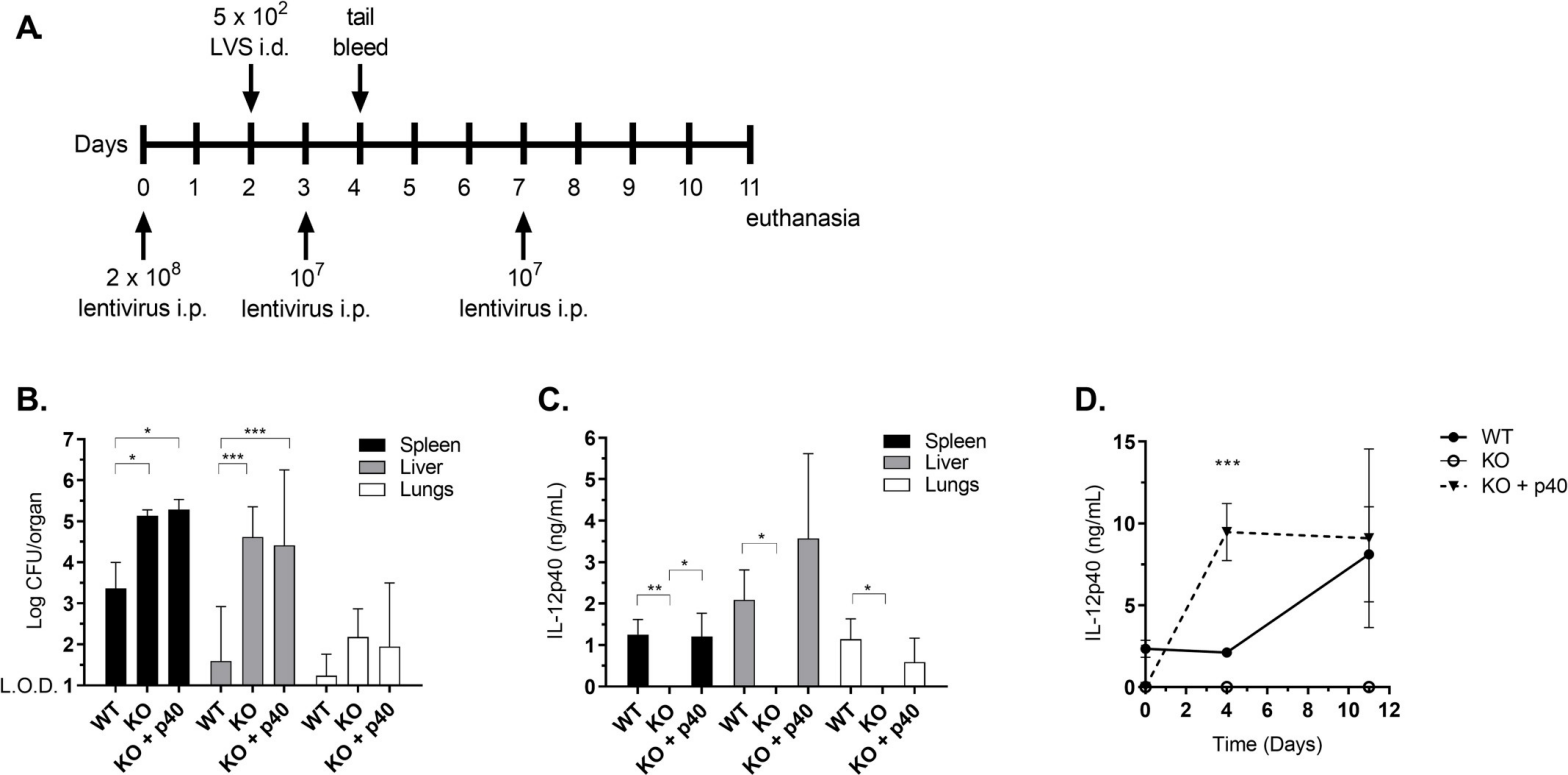
Administration of IL-12p40-overexpressing lentivirus does not rescue p40 KO mice from chronic infection. The indicated mice were given 2 x 10^8^ pHAGE-Il12b2 lentivirus or PBS i.p. on day 0, then challenged with 5 x 10^2^ LVS or PBS i.d. on day 2 (A). Mice were then given two additional treatments of 10^7^ lentivirus or PBS i.p. at days 3 and 7. Bacterial burdens were determined on day 11 (B) and IL-12p40 expression was determined by ELISA in spleen, liver, lungs (C), and sera (D). Data are from a representative of five independent experiments of similar design. Mice used were ages 4–9 months and were aged matched within each experiment. * p < 0.05; ** p < 0.01; *** p < 0.001 between KO + p40 and WT by ANOVA with Tukey’s multiple comparisons test.

To test whether reconstitution of IL-12p40 levels before LVS exposure enabled p40 KO mice to clear infection, mice were given one lentiviral treatment with the maximal practical dose two days before sublethal LVS infection; to sustain maximal IL-12p40 levels, mice received two additional lentiviral treatments periodically after LVS infection (Fig 3A). At the experimental endpoint, WT mice had begun to control the infection (Fig 1 and Fig 3B), whereas spleens and livers of p40 KO mice contained significantly more bacteria. No difference in bacterial burdens between p40 KO mice that received lentiviral treatment and those that did not was detected, however, despite the presence of high levels of IL-12p40 protein quantitated by ELISA in both organ homogenates and sera from treated mice (Fig 3C and 3D). Analyses of splenocyte subpopulations by flow cytometry at the experimental endpoint (day 11) did not reveal any significant differences in the proportions of lymphocytes between p40 KO mice receiving PBS compared to those receiving lentivirus, although proportions of neutrophil and macrophage populations trended lower in the spleens of lentivirus-treated mice compared to infected WT and untreated infected KO mice (S4 Fig). Absolute numbers of B and T cells were significantly decreased in spleens of lentivirus-treated mice indicating possible slight changes in the recruitment of some lymphocyte populations and potentially some biological impact of exogenously delivered p40. However, collectively the data indicate that direct treatment with these forms of IL-12p40 protein does not rescue p40 KO mice from chronic *F*. *tularensis* infection.

## Discussion

Driven by previous findings that identified IL-12p40 as critical for the development of innate and adaptive responses to *F*. *tularensis*, we further investigated the role of this cytokine in clearance of LVS primary vaccinating infection. Upon low dose LVS vaccination, mice deficient in IL-12p40 develop a chronic infection that is not lethal but does not resolve. However, similar to WT mice, IL-12p35-deficient mice eliminated primary LVS infection despite low levels of IFN-γ, a critical cytokine for survival and clearance of many intracellular pathogens, including *F*. *tularensis* [22, 34]. Although suboptimal IFN-γ production due to lack of IL-12p70 may contribute to the infection phenotype seen in p40 KO mice, p40 clearly functions during LVS clearance independently of heterodimeric IL-12 or IL-23 [22, 23]. Here we demonstrated that primed splenocytes from p40 KO and p35 KO mice have comparable, if not better, abilities to control intramacrophage LVS growth *in vitro*, even with similar low IFN-γ expression. We further identified a panel of genes that were strongly expressed by WT and p35 KO splenocytes recovered from *in vitro* co-cultures but exhibited much reduced expression in p40 KO splenocytes; as expected, many of these genes are related to the regulation of IFN-γ. While some are candidates for future study, none suggested obvious explanations for the p40 defect. We therefore directly evaluated a role for p40 itself. We found that reconstitution of p40 KO mice with IL-12p40 monomer and/or homodimer either before or during LVS infection did not induce LVS clearance.

The course of primary LVS infection, particularly at 7 and 10 days, suggested a delay and ultimately an inability of adaptive immune responses to clear LVS infection in p40 KO mice. However, the studies presented previously [22] and extended here to directly compare relative frequencies of responding splenocytes do not support this idea. Using an *in vitro* functional assay, we confirmed that splenocytes from LVS-primed p40 KO mice were as functional as those from WT and p35 KO cells. Of note, because T cells are the primary cell that effects control of intramacrophage LVS growth in this *in vitro* co-culture system [35], these results imply that the T cell arm of adaptive immunity to LVS is not obviously impaired in p40 KO mice. Results are also unlikely to be related to gross deficiencies in humoral immunity, because previous results demonstrated that sera from LVS-infected IL-12p40 KO mice had notably increased quantities of anti-LVS antibodies compared to LVS-infected C57BL/6J or IL12p35 KO mice [22, 23]. Additionally, we observed that both primed p40 KO and p35 KO splenocytes expressed minimal IFN-γ upon *in vitro* re-stimulation, which is consistent with previous reports [13, 22]. Therefore, the lack of *Francisella* clearance in p40 KO mice cannot be attributed to reduced production of IFN-γ alone. Previous studies have shown that although IFN-γ is critical for immunity to *F*. *tularensis* infection, IFN-γ alone is not necessarily sufficient and does not reliably predict survival in mice [26, 37].

Mechanisms of IFN-γ-independent immunity have been seen in studies with other intracellular infections where IFN-γ is known to be critical for resistance, such as *M*. *tuberculosis*. For example, IFN-γ-producing CD4^+^ T cells are essential for protection against *M*. *tuberculosis* infection in mice and humans [38, 39]; however, adoptive transfer of both WT and IFN-γ KO activated T cells limited lung CFU in recipient *M*. *tuberculosis*-infected, IFN-γ KO mice [40]. Another recent study found that mice given IFN-γ-neutralizing antibody during secondary *L*. *monocytogenes* challenge had similar bacterial burdens 3 days after infection compared to untreated mice [41]. Our data also continues to support an IFN-γ-independent, IL-12p40-dependent mechanism of clearance in *F*. *tularensis* infection.

NO-independent mechanisms may also play a role in the response to LVS. NO production is important for the survival of LVS infection in mice and has been demonstrated using several mouse models, including aerosol infection in iNOS KO mice [42]. iNOS KO are highly susceptible to LVS aerosol infection and succumb to low doses within 2 weeks. Additionally, TNF KO mice which have a deficit in NO, display higher bacterial burdens after a low dose i.d. LVS infection compared to WT mice [43]. Primed splenocytes from p40 KO mice can clearly induce the production of high levels of NO and are able to control intramacrophage bacterial growth in vitro to a similar degree as WT and p35 KO cells, yet cannot clear the infection in vivo. While NO production in p40 KO mice may contribute to the initial control of in vivo infection, it is likely that NO-independent mechanisms are involved in ultimate bacterial clearance.

In order to search for non-IFN-γ-dependent mechanisms by which p40 expression contributes to LVS clearance, we screened expression of a panel of inflammatory and cytokine genes in *in vitro* re-stimulated splenocytes recovered from co-cultures. Gene expression data from recovered cells reflect activities in lymphocytes, particularly T cells [6]. We identified a subset of genes that were upregulated in WT or p35 KO splenocytes but not in p40 KO splenocytes. Some of these genes, including IL-12rβ2, TNF-α, T-bet, and GM-CSF, have been previously identified as potential correlates of protection against *F*. *tularensis* infection using functional assays with murine splenocytes or peripheral blood lymphocytes [44]. The Th1-specific transcription factor T-bet is upregulated by IL-12 or IFN-γ signaling through STAT4 [45]. T-bet has been shown to be important for host resistance to primary LVS infection, particularly in the lungs, and T-bet KO splenocytes from LVS-vaccinated mice exhibit impaired ability to control intramacrophage LVS in co-culture [46]. While most of the targets we identified are well-established mediators of inflammation through the induction IFN-γ, our data suggest these factors may have additional IL-12p40/p80-dependent activities that are unrelated to IFN-γ production.

Other genes identified here that are not as directly related to Th1 responses and IFN-γ, such as IL-13, IL-9, and IL-17a, are candidates for further study. Some of these targets have been studied in connection to host resistance to primary infection with other intracellular bacteria, such as *M*. *tuberculosis* [47–49]. However, their roles in *F*. *tularensis* clearance, particularly in the context of IL-12p40/p80 signaling, are not yet studied or are not clear. For example, IL-23-dependent IL-17 production has been implicated in resistance to pulmonary LVS infection in mice. After intratracheal challenge with LVS, IL-23p19 KO mice had higher bacterial burdens in lungs, along with decreased IL-17 levels and numbers of IL-17-producing cells [50]. Despite this, IL-23 was largely dispensable for immunity to i.d. or i.n. LVS infection [23]. Therefore, this gene set represents candidates that deserve further exploration of possible IFN-γ-independent roles in mediating intracellular bacterial infection clearance through IL-12p40/p80. These results collectively provide the impetus to perform unbiased screens in the future, such as by applying RNAseq.

To directly evaluate a potential mechanism for p40 protein in *F*. *tularensis* clearance, we used two methods to reconstitute IL-12p40 levels in p40 KO mice throughout the course of LVS primary infection. One approach used intermittent injection of purified p40 homodimer (p80) after LVS infection was established, while the second strategy used a lentivirus construct to continuously deliver p40 before and during LVS infection. Disappointingly, treatment of KO mice by either approach yielded readily detectable levels of IL-12p40 in sera and spleens but did not reduce chronic LVS infection. Of note, our data demonstrate that p40 levels in the spleen, liver, lungs, and sera of KO mice given recombinant lentivirus were consistently similar to or higher than levels in WT control mice. The interpretation that treatments achieved physiological levels is further supported by the observation that we detected sera levels comparable to those observed in previous studies [22, 51, 52]. Here, serum p40 levels in WT mice ranged from 2.5 to 12 ng/mL throughout 9 days after sublethal infection. Previous studies have reported serum p40 levels in C57BL/6J mice ranging 2.5–25 ng/mL throughout 28 days after sublethal i.d. LVS infection, and 1–2 ng/mL during 1–3 weeks after i.v. injection with M. bovis BCG [22, 51]. Our experiments were terminated within two weeks of infection and could be extended; however, the lack of measurable impact of p40 treatment on any parameter did not justify further use of animals for such extended studies.

This study has limitations that impact interpretations of results in which exogenous p40 treatment did not impact LVS clearance. First, p40 monomer and homodimer may serve separate functions in LVS clearance that these approaches did not fully recapitulate. This possibility is difficult to test experimentally because depletion of p40 in mice also removes p80. Second, autocrine or paracrine roles for p40 may not be recapitulated with delivery of exogenous p40. Third, our approaches may not have provided sufficient levels of p40. Studies with *M*. *tuberculosis*, *L*. *major*, and other intracellular pathogens have used extended treatment of mice with recombinant p40 homodimer (p80); however, most models use daily dosing with microgram quantities of recombinant protein, which may be too high to be physiologically relevant [31, 53]. Our treatment methods achieved delivery of levels of p40 protein in p40 KO mice similar to those observed in LVS-infected WT mice. Finally, we also cannot rule out the possibility that these treatment strategies did not achieve appropriate circulation of sufficient p40 levels *in vivo* to approximate those available during LVS infection of WT mice. However, we believe that this possibility is unlikely. Instead, we consider these results to support the continued search for as-yet undiscovered functions of precisely localized p40 that indirectly lead to bacterial clearance.

To date, intrinsic functional activities of IL-12p40 have not been fully characterized. IL-12p40 monomers and homodimers (p80) have been suggested to have individual agonistic functions separate from IL-12 and IL-23 that can mediate robust immunostimulatory or immunosuppressive responses. Both subunits may also act as an antagonist by blocking IL-12R in some contexts. Signaling activities induced by p40 and p80 independently of IL-12 or IL-23 are also not fully defined, but it is likely that these proteins can independently promote different cellular responses. One recent example reported detrimental agonistic activities of p80 in exacerbating inflammation and disease during H5N1 influenza virus infection in mice [19]. These studies found that the majority of total IL-12p40 in the bronchoalveolar lavage fluid of H5N1-infected mice was in the form of p80 homodimer, and specific antibody blockade of p80 ameliorated weight loss and reduced cellular infiltration in the lungs of H5N1-infected mice. In the context of intracellular infections such as murine infection with *M*. *tuberculosis*, p80 is also thought to have protective agonistic functions (in addition to those of IL-12p70). DC in p40 knockout (KO) mice failed to migrate from the lungs to the draining lymph node following exposure to *M*. *tuberculosis*, which correlated with significantly less antigen-specific IFN-γ-producing CD4^+^ T cells in lungs [16]. However, *in vivo* DC migration and T cell activation was rescued when activated p40 KO bone marrow-derived DC were treated *ex vivo* with p80, but not p70, before intratracheal delivery to the lung.

On the other hand, immunosuppressive activities of p80 have been reported in models of murine colitis. One such study observed that treatment with either recombinant p80 or with a HIF-stabilizing drug treatment that induces p80 expression suppressed IFN-γ^+^ Th1 and Th17 lymphocyte populations and was protective against colitis in mice [54]. Also of note, much less is known about the functions of p40 as a monomer. A recent study linked p40 monomer to the attenuation of autoimmune signaling in multiple sclerosis (MS) via suppression of IL-12Rβ1 internalization in a murine model (EAE) as well as in human patients [55]. Collectively, these studies and others indicate p40 and p80 effect independent responses that can be protective or cause damaging tissue inflammation depending on the context of disease. The balance between protective versus host-detrimental effects of p40/p80 activities may be the key to regulating clearance of *F*. *tularensis* LVS.

Taken together, we believe the present studies indicate that circulating p40 monomers/dimers alone are not directly responsible for effecting clearance of LVS. Because LVS is a prototypical intracellular bacterium, the mechanism involved remains an important topic for future study. The gene candidates identified here, coupled with similar broader unbiased screens, provide a logical starting point to seek other mediators that are involved. Other candidates are further suggested by a recent study in which affinity purification from the serum of LPS-challenged p35 KO mice revealed over 20 novel binding partners of p40 that could potentially bind extracellularly [56]. The functions and physiological relevance of these complexes have yet to be defined and merit further investigation in the context of murine LVS infection. Future studies will therefore focus on downstream effectors that are activated (or repressed) by appropriately localized p40 production.

## Supporting information

S1 Fig Chronically infected IL-12p40 KO mice show immune dysregulation in the spleen. Mice were infected with 10^5^ LVS i.d. Three mice per group were sacrificed at 3, 7, 10, 20, and 30 days post-infection to assess splenocyte subpopulations by flow cytometry. Data shown are proportions of live CD45^+^ cells per spleen (A) and absolute cell number per spleen (B) from a representative experiment of two independent experiments. Mice used were ages 2–6 months were aged matched within each experiment. *p < 0.05; ** p < 0.01; *** p < 0.001 by Holm-Šídák multiple t-test between WT and KO samples.
(TIF)

S2 Fig Exogenous IL-12p80 treatment does not resolve chronic infection in IL-12p40 KO mice. Mice aged 2–6 months were infected with 10^5^ LVS i.d. Beginning 31 days after infection, five mice per group were given either 50 μg of recombinant IL-12p80 or PBS i.p. every other day for seven days, after which bacterial burdens were assessed (A). IL-12p40 was readily detected in organ homogenates from p40 KO mice treated with IL-12p80 (B). Data shown are from a single experiment. *** p < 0.001 by Holm-Šídák multiple t-test.
(TIF)

S3 Fig IL-12p40 is detectable in sera of IL-12p40 KO mice treated with an overexpression vector. The coding sequence of murine IL-12p40 was inserted into the lentiviral plasmid pHAGE-CMV-MCS-IRES-ZsGreen-W to create a lentiviral expression construct that overexpresses IL-12p40 protein (A). HEK293T cells were transfected with the plasmid (B) or transduced with decreasing MOIs of lentivirus containing the plasmid (C). After 48 h of culture, supernatants were collected for analyses of IL-12p40 expression by ELISA, and cells were purified for protein analysis by western blot (D). Mice were bled at days 3, 7, 11, 15, 20, and 27 after treatment with the indicated dose of lentivirus i.p. at day 0. Sera were collected at days 3, 7, 11, 15, 20, and 27 after treatment with the indicated dose of lentivirus i.p. at day 0 and analyzed for IL-12p40 expression by ELISA (E). IL-12p40 in spleen homogenates at day 27 was below the limit of detection. The results shown are from one representative of three independent experiments of similar design. Mice aged 6–24 months were aged matched within each experiment. * p < 0.05 compared to pHAGE-Empty treatment by Holm-Šídák multiple t-test.
(TIF)

S4 Fig Splenocyte subpopulations following lentivirus administration and subsequent LVS infection. KO mice received lentivirus administrations and a sublethal dose of LVS as detailed in Fig 3A. On day 11, splenocytes from five mice per group were pooled and stained with the indicated markers for flow cytometry. Data shown are proportions of live CD45^+^ cells per spleen (A) and absolute cell number per spleen (B) from two independent experiments. * p < 0.05; ** p < 0.01; *** p < 0.001 by ANOVA with Tukey’s multiple comparisons test against infected samples.
(TIF)

S1 Table Gene expression profiling of splenocytes following co-culture. Splenocytes from naïve mice, or mice vaccinated with 10^5^ LVS i.d., were co-cultured with LVS-infected BMDMs from the same genotype. Splenocytes were collected from co-culture two days after infection and used to purify RNA. Gene expression was analyzed by RT-PCR using probes against a panel of 84 inflammatory cytokine and chemokine genes. Values shown are represented as fold changes over genotype-matched naïve samples. Data are the average fold changes from three independent experiments. Mice used were ages 3–9 months were aged matched within each experiment. * p < 0.05 between KO and WT; # p < 0.05 between p40 KO and p35 KO by ANOVA with Tukey’s multiple comparisons test.
(TIF)

S1 Raw images (PDF)
