# Supplementary figures and images for: IL-12p40 is essential but not sufficient for Francisella tularensis LVS clearance in chronically infected mice
Source: PLoS One. 2023 Mar 27;18(3):e0283161. doi: 10.1371/journal.pone.0283161 (PMC10042368; doi:10.1371/journal.pone.0283161)

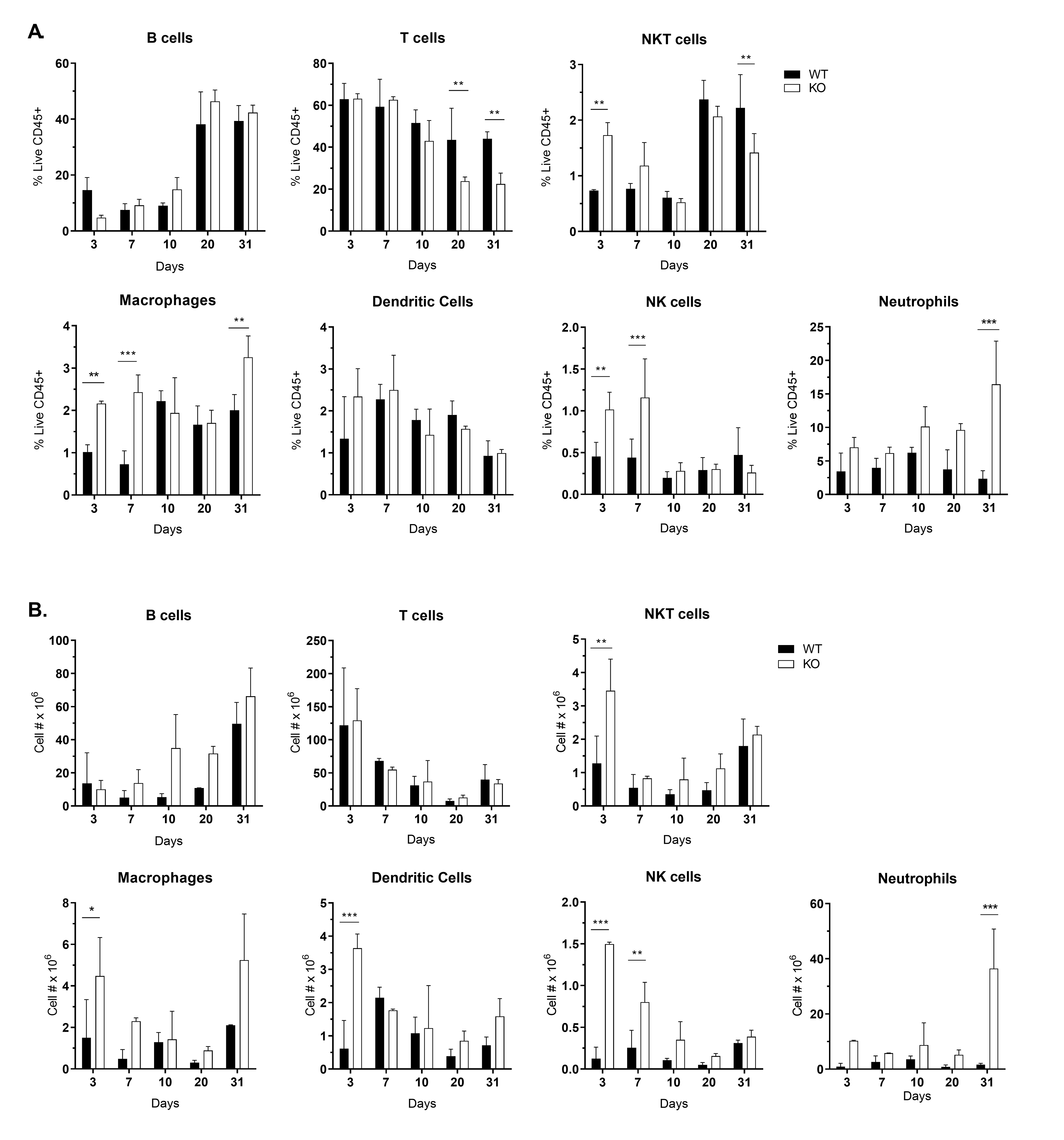

Supplement: S1 Fig — Mice were infected with 105 LVS i.d. Three mice per group were sacrificed at 3, 7, 10, 20, and 30 days post-infection to assess splenocyte subpopulations by flow cytometry. Data shown are proportions of live CD45+ cells per spleen (A) and absolute cell number per spleen (B) from a representative experiment of two independent experiments. Mice used were ages 2–6 months were aged matched within each experiment. *p < 0.05; ** p < 0.01; *** p < 0.001 by Holm-Šídák multiple t-test between WT and KO samples. (TIF) [file pone.0283161.s001.tif]

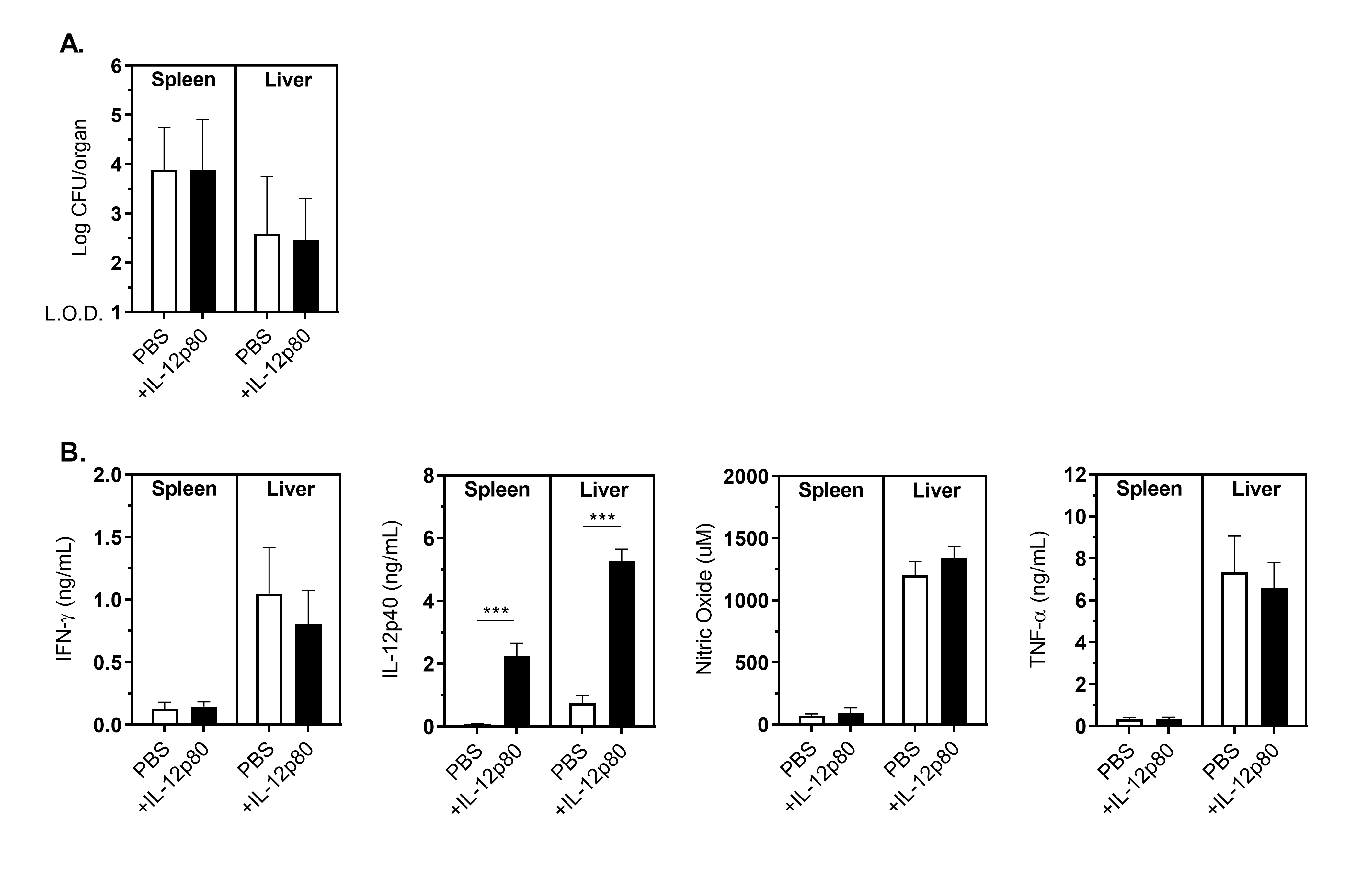

Supplement: S2 Fig — Mice aged 2–6 months were infected with 105 LVS i.d. Beginning 31 days after infection, five mice per group were given either 50 μg of recombinant IL-12p80 or PBS i.p. every other day for seven days, after which bacterial burdens were assessed (A). IL-12p40 was readily detected in organ homogenates from p40 KO mice treated with IL-12p80 (B). Data shown are from a single experiment. *** p < 0.001 by Holm-Šídák multiple t-test. (TIF) [file pone.0283161.s002.tif]

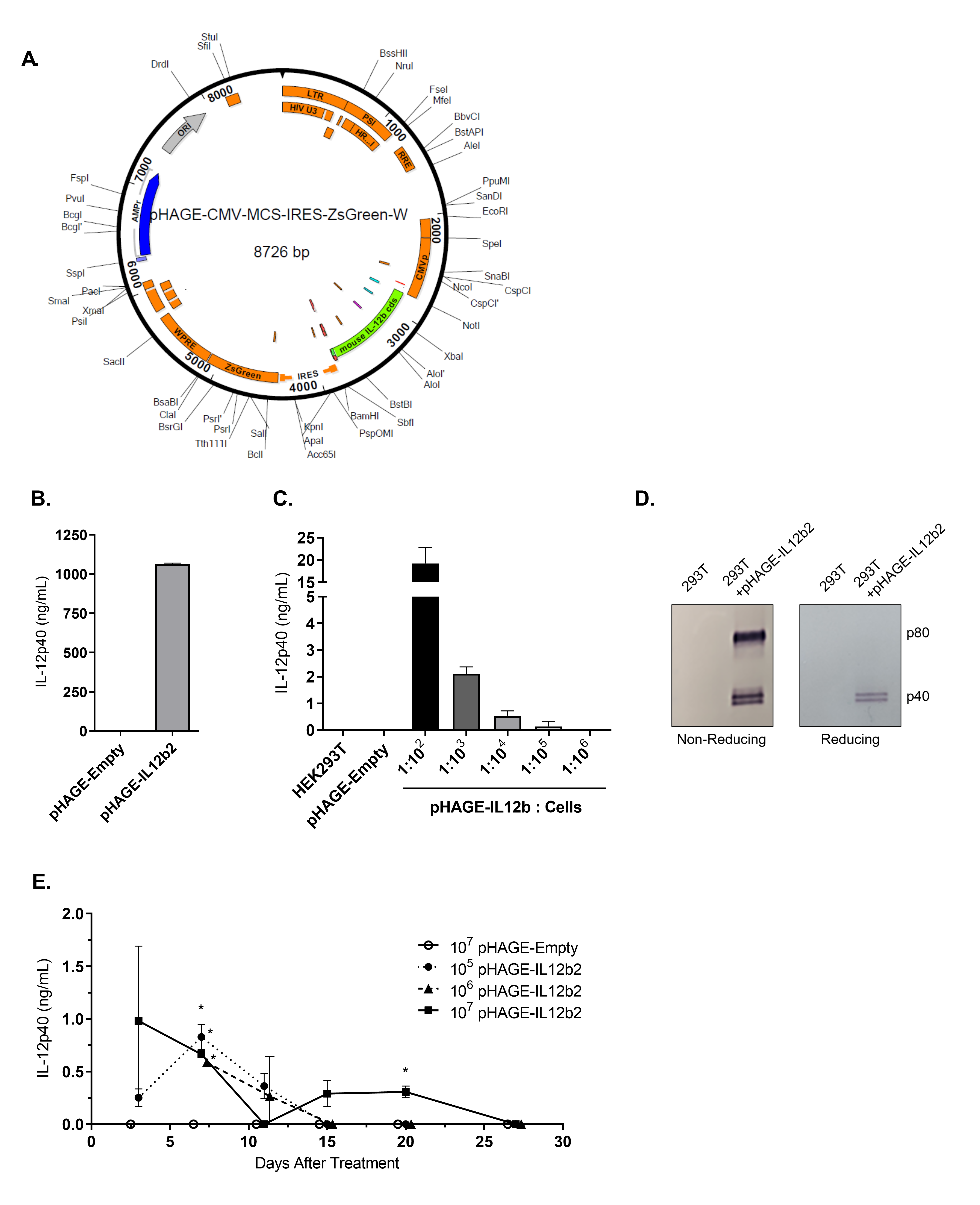

Supplement: S3 Fig — The coding sequence of murine IL-12p40 was inserted into the lentiviral plasmid pHAGE-CMV-MCS-IRES-ZsGreen-W to create a lentiviral expression construct that overexpresses IL-12p40 protein (A). HEK293T cells were transfected with the plasmid (B) or transduced with decreasing MOIs of lentivirus containing the plasmid (C). After 48 h of culture, supernatants were collected for analyses of IL-12p40 expression by ELISA, and cells were purified for protein analysis by western blot (D). Mice were bled at days 3, 7, 11, 15, 20, and 27 after treatment with the indicated dose of lentivirus i.p. at day 0. Sera were collected at days 3, 7, 11, 15, 20, and 27 after treatment with the indicated dose of lentivirus i.p. at day 0 and analyzed for IL-12p40 expression by ELISA (E). IL-12p40 in spleen homogenates at day 27 was below the limit of detection. The results shown are from one representative of three independent experiments of similar design. Mice aged 6–24 months were aged matched within each experiment. * p < 0.05 compared to pHAGE-Empty treatment by Holm-Šídák multiple t-test. (TIF) [file pone.0283161.s003.tif]

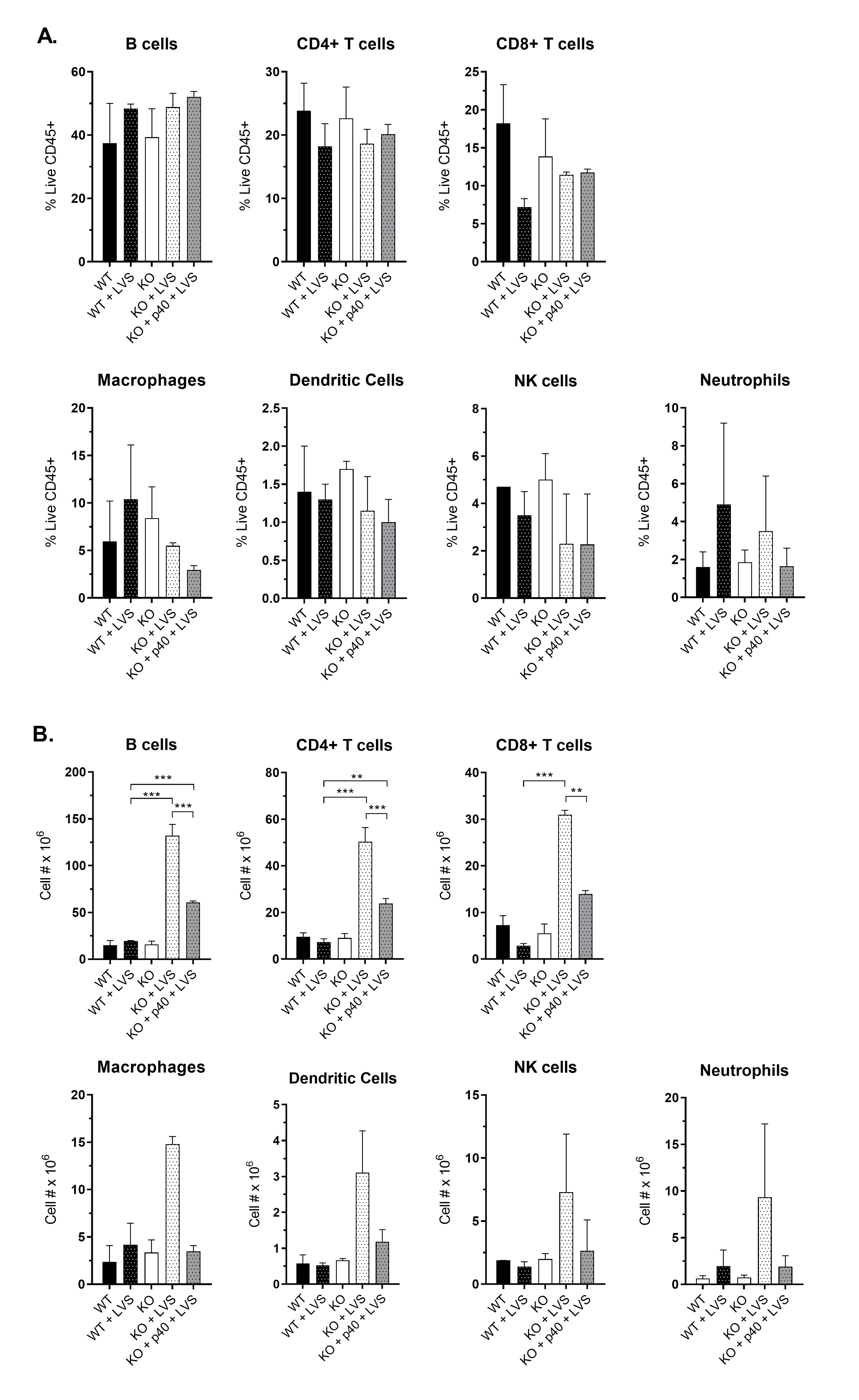

Supplement: S4 Fig — KO mice received lentivirus administrations and a sublethal dose of LVS as detailed in Fig 3A. On day 11, splenocytes from five mice per group were pooled and stained with the indicated markers for flow cytometry. Data shown are proportions of live CD45+ cells per spleen (A) and absolute cell number per spleen (B) from two independent experiments. * p < 0.05; ** p < 0.01; *** p < 0.001 by ANOVA with Tukey’s multiple comparisons test against infected samples. (TIF) [file pone.0283161.s004.tif]

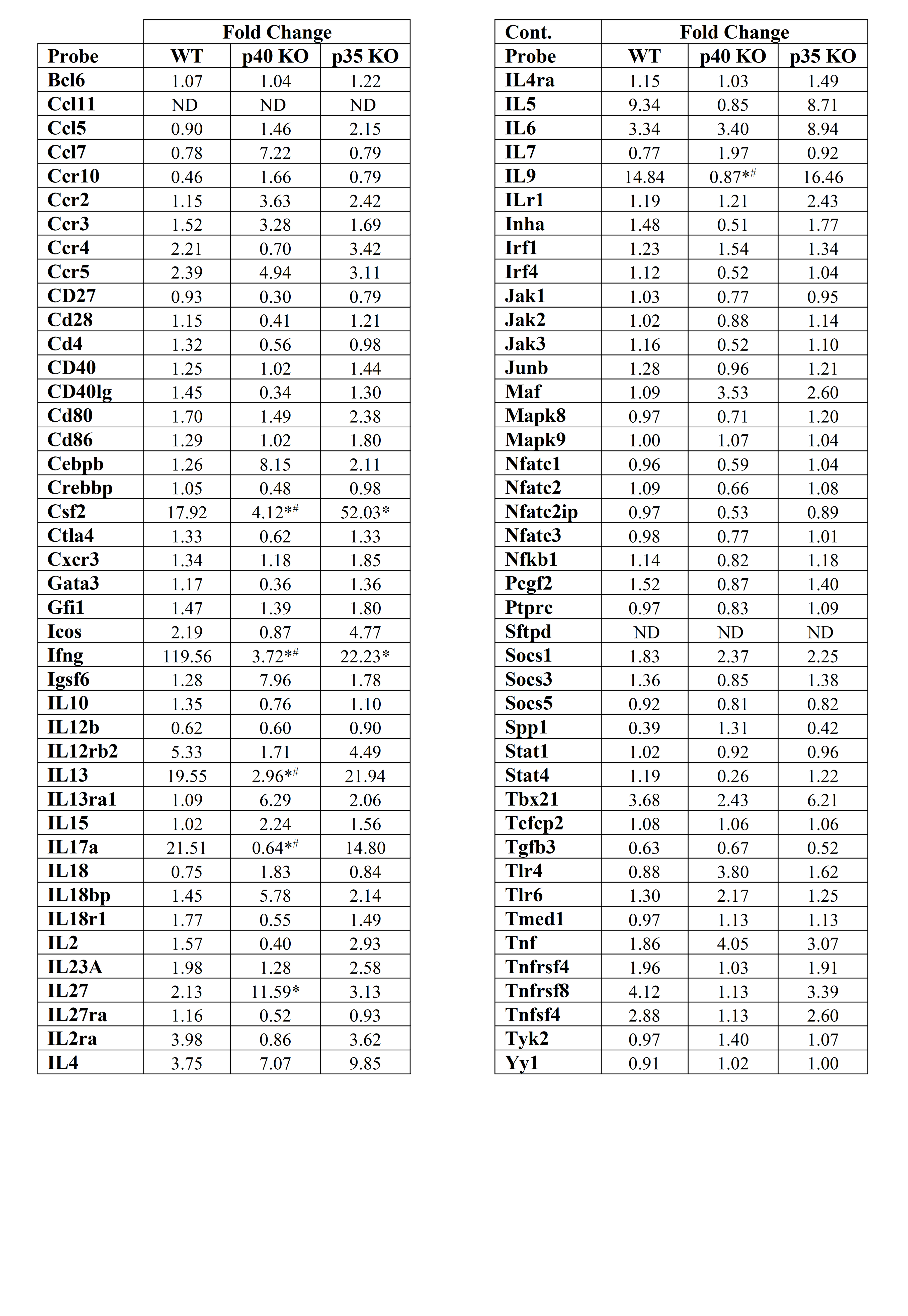

Supplement: S1 Table — Splenocytes from naïve mice, or mice vaccinated with 105 LVS i.d., were co-cultured with LVS-infected BMDMs from the same genotype. Splenocytes were collected from co-culture two days after infection and used to purify RNA. Gene expression was analyzed by RT-PCR using probes against a panel of 84 inflammatory cytokine and chemokine genes. Values shown are represented as fold changes over genotype-matched naïve samples. Data are the average fold changes from three independent experiments. Mice used were ages 3–9 months were aged matched within each experiment. * p < 0.05 between KO and WT; # p < 0.05 between p40 KO and p35 KO by ANOVA with Tukey’s multiple comparisons test. (TIF) [file pone.0283161.s005.tif]
