# Supplementary material for: IL-12p40 is essential but not sufficient for Francisella tularensis LVS clearance in chronically infected mice
Source: PLoS One. 2023 Mar 27;18(3):e0283161. doi: 10.1371/journal.pone.0283161 (PMC10042368; doi:10.1371/journal.pone.0283161)
Supplement: S1 Raw images — (PDF) [file pone.0283161.s006.pdf]

Supporting Information for Figure S3

Uncropped Non-Reducing Western Blot Image

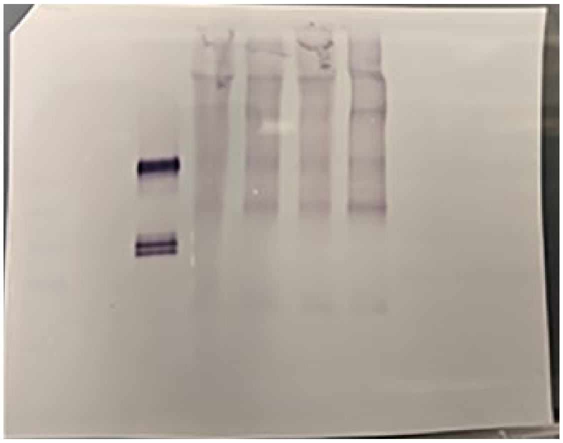

Lane	Sample	Used in Figure S3
1	Ladder	No
2	293T	Yes
3	293T + pHAGE-IL12b2	Yes
4	KO spleen homogenate	No
5	KO + pHAGE spleen homogenate	No
6	KO + pHAGE spleen homogenate	No
7	C57 spleen homogenate	No

Uncropped Reducing Western Blot Image

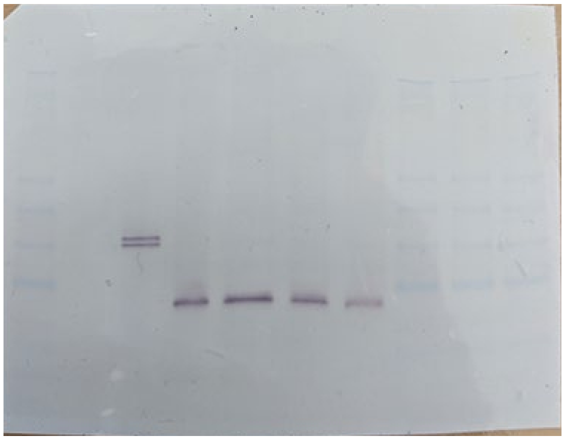

Lane	Sample	Used in Figure S3
1	Ladder	No
2	293T	Yes
3	293T + pHAGE-IL12b2	Yes
4	KO splenocytes	No
5	KO + pHAGE splenocytes	No
6	KO + pHAGE splenocytes	No
7	C57 splenocytes	No
8	Ladder	No
9	Ladder	No
10	Ladder	No
